# The properties of bioengineered chondrocyte sheets for cartilage regeneration

**DOI:** 10.1186/1472-6750-9-17

**Published:** 2009-03-06

**Authors:** Genya Mitani, Masato Sato, Jeong IK Lee, Nagatoshi Kaneshiro, Miya Ishihara, Naoshi Ota, Mami Kokubo, Hideaki Sakai, Tetsutaro Kikuchi, Joji Mochida

**Affiliations:** 1 Department of Orthopaedic Surgery, Surgical Science, Tokai University School of Medicine, Kanagawa, Japan; 2 Department of Medical Engineering, National Defense Medical Collage, Saitama, Japan; 3 CellSeed Inc., Tokyo, Japan

## Abstract

**Background:**

Although the clinical results of autologous chondrocyte implantation for articular cartilage defects have recently improved as a result of advanced techniques based on tissue engineering procedures, problems with cell handling and scaffold imperfections remain to be solved. A new cell-sheet technique has been developed, and is potentially able to overcome these obstacles. Chondrocyte sheets applicable to cartilage regeneration can be prepared with this cell-sheet technique using temperature-responsive culture dishes. However, for clinical application, it is necessary to evaluate the characteristics of the cells in these sheets and to identify their similarities to naive cartilage.

**Results:**

The expression of SOX 9, collagen type 2, 27, integrin α10, and fibronectin genes in triple-layered chondrocyte sheets was significantly increased in comparison to those in conventional monolayer culture and in a single chondrocyte sheet, implying a nature similar to ordinary cartilage. In addition, immunohistochemistry demonstrated that collagen type II, fibronectin, and integrin α10 were present in the triple-layered chondrocyte sheets.

**Conclusion:**

The results of this study indicate that these chondrocyte sheets with a consistent cartilaginous phenotype and adhesive properties may lead to a new strategy for cartilage regeneration.

## Background

Osteoarthritis (OA), the most common articular disorder, is characterized primarily by slow progressive degeneration or destruction of cartilage. However, the exact etiology of OA is not known. The symptoms of osteoarthritis usually appear in middle age and almost everyone has them by age 70. Therefore, adequate treatments for the early stages of degeneration are required.

Cartilage has two important functions, the reduction of friction and the transmission of load. Some of the specific properties of cartilage are a lack of blood vessels, a small number of cell constituents, and a large amount of extracellular matrix (ECM). Once cartilage has been damaged, it is unable to heal itself.[1,2] There are various treatments for damaged cartilage, but few recommended surgical procedures. Drilling, subchondral abrasion[3] and microfracture treatments[4] allow the regeneration of damaged cartilage by activating mesenchymal stem cells derived from the bone marrow; however, previous reports have shown that the regenerated cartilage was fibrocartilage, not hyaline cartilage. The functions and properties of fibrocartilage are inferior to hyaline cartilage, and therefore the outcomes at long-term follow-up after these treatments tend to be poor.[2] Mosaicplasty can be used to transplant hyaline cartilage to the damaged area and reports have shown at long-term follow-up that mosaicplasty is beneficial; however, it has associated donor site morbidity, and only a predetermined defect area can be treated.[5] The clinical results of arthroplasty for severe osteoarthritis have improved with the development of new surgical techniques and the selection of appropriate medical devices. However, many obstacles have yet to be overcome, including limited range of motion and durability, and excessive invasiveness of the surgery. In addition, resulting function is significantly inferior to that of the normal joint. Therefore, the establishment of new protocols for cartilage regeneration using tissue engineering is important. Because of recent progress in tissue engineering, various techniques are available to cure damaged cartilage. Autologous chondrocyte implantation (ACI), first reported by Brittberg *et al*., [6] has been used clinically. Although clinical results show that this technique can be beneficial, some problems remain, such as limits on the size of lesions that can be treated, periostal hypertrophy, and the lack of appropriate methods to evaluate the regenerated cartilage after ACI. Moreover, although the clinical results of ACI have recently improved as a result of advanced techniques based on tissue engineering procedures, problems relating to cell handling and scaffold imperfections remain. Artificial scaffolds have been adopted to deliver cells into cartilage defect sites, and to reinforce the mechanical stability of three-dimensional tissue engineered chondral grafts. The ideal scaffold is supposed to encourage ECM. Although, some scaffolds have been successfully applied for the cartilage regeneration,[7] there are problems with biocompatibility and cellular viability, including cell attachment, distribution and proliferation.

Recently, a cell-sheet technique[8] has been developed that is potentially able to overcome these problems. Therefore, a new strategy for cartilage regeneration without a scaffold has been studied with cell-sheet technology using temperature-responsive culture dishes (UpCell™ CellSeed Inc., Tokyo, Japan).

We previously reported[9] the implantation of layered chondrocyte sheets, harvested by simply lowering the temperature and with no need for enzyme digestion, in Japanese white rabbits. We also verified the effectiveness of chondrocyte sheets using a swine partial cartilage defect model, which showed reduced degeneration. Interestingly, in layered chondrocyte sheets, it appeared that catabolic factors such as MMP3, MMP13, and ADAMTS5 decrease at the point of layering, while the expression of TIMP1, an inhibitor of MMP3, increases.[9] This indicates that layered chondrocyte sheets have fewer destructive factors than degenerate cartilage and have good adhesion properties, which help to both protect and repair the cartilage surface.[9] However, the precise mechanisms by which such chondrocyte sheets adhere to the damaged cartilage and maintain the cartilage phenotype remain to be elucidated. The purpose of this study was to further investigate the properties of human chondrocyte sheets using scanning electron microscopic evaluation and gene expression and immunohistochemical analyses.

## Results

### Manipulation of chondrocyte sheets

**Figure 1:**
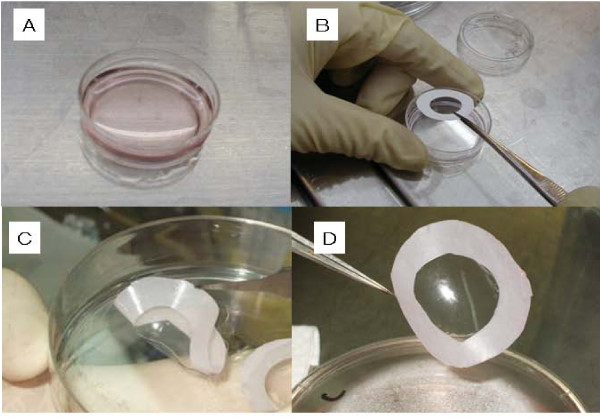
**Fabrication of the cell sheets**. Temperature-responsive culture dishes on which chondrocytes had been cultured were removed from the incubator when the cells reached confluence and were let stand at about 25°C for 30 min (A). After the culture medium was removed, a polyvinylidene difluoride (PVDF) membrane was put onto the dish (B), and the sheet was detached gently (C). The chondrocyte sheets could then be easily fabricated into multilayered constructs with the help of the PVDF and without the need for enzyme digestion (D).

Chondrocyte sheets prepared as either cell monolayer sheets or three-layered sheets were obtained by simply reducing the temperature, with no need for an enzymatic digestion step (Fig. 1). The chondrocytes were harvested as a single contiguous cell sheet, retaining the neighboring extracellular structure, which implies that these cell sheets should contain extracellular proteins including cell-cell junction, ECM, and adhesion proteins.

The multilayered sheets could be easily produced by placing one chondrocyte sheet onto other sheets by making use of the supporting PVDF membrane (Fig. 1A–C). By repeating this procedure twice, three-layered cell sheets were obtained (Fig. 1D). When cultured for 1 week, the triple-layered chondrocyte sheets were extendable and were not damaged by mild external force. This extended multilayering process was sufficient to give a single contiguous multilayered structure in which each sheet had adhered firmly and tightly to the other sheets.

With the help of the supporting membrane, the cell-sheet-PVDF film showed good stability and we could easily handle the chondrocyte sheets.

### Scanning electron microscopy

**Figure 2:**
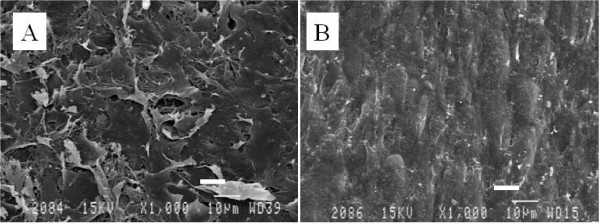
**Scanning electron microscopy**. Scanning electron microscopy revealed that the top (A) and the basal aspects (B) of the chondrocyte sheet demonstrated completely different textures. The chondron like texture was only observed on the basal aspect, which had adhesive properties. Scale bar = 10 μm.

SEM analysis revealed that the top and basal aspects of the chondrocyte sheets showed completely different textures. A network of laminated ECM structures was observed on the top aspect of the sheet. These sheets of ECM structures appeared piled up, with several sheet-like configurations and amorphous shapes, and separated edges of the ECM sheet occasionally being observed as dog-ears facing the culture medium side (Fig. 2A).

The surface of the basal aspect, which had been attached to the bottom of the culture dish, was covered with a smooth ECM pattern, and numerous humps (mound-like elevations) were observed. Compared with the top side of the chondrocyte sheet, the arrangement of the accumulated ECM surface was smoother, with a parallel pattern (Fig. 2B).

### Analysis of gene expression

**Figure 3:**
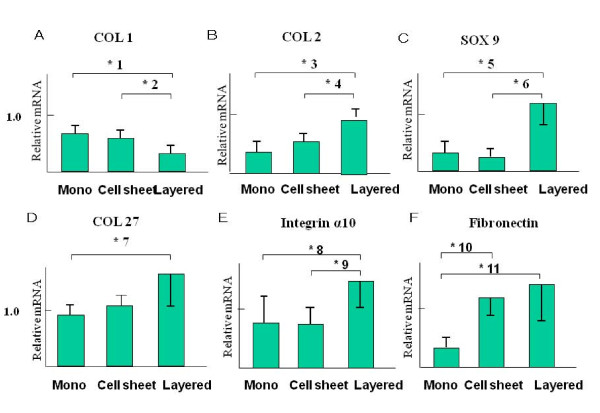
**Relative expression of mRNA**. The y-axis shows the mRNA expression relative to that of glyceraldehyde-3-phosphate dehydrogenase (GAPDH). The results were evaluated using the SmartCycler II software program. GAPDH expression was used to normalize samples. The error bars represent the standard deviation. Type I collagen mRNA expression was present at a low level in the layered chondrocyte cell sheets (A). In contrast, type II collagen mRNA expression was at significantly higher levels in the layered chondrocyte sheets in comparison to the monolayer cultures and monolayer chondrocyte sheets (B). SOX9 and COL27 mRNA expression were observed at significantly higher levels in the layered chondrocyte sheets in comparison to conventional monolayer cultures and monolayer chondrocyte sheets (C, D). Integrin α10 and fibronectin mRNA expression were at significantly higher levels in the layered chondrocyte sheets in comparison to the conventional monolayer cultures and the monolayer chondrocyte sheets (E, F).

The expression of collagen type 1 (COL1) mRNA was observed at significantly lower levels in the layered chondrocyte sheets in comparison to the conventional monolayer cultures and monolayer chondrocyte sheets (Fig. 3A).

In contrast, the expression of collagen type 2 (COL2), SOX9, COL 27, integrin α10 and fibronectin mRNAs were observed at significantly higher levels in the layered chondrocyte sheets in comparison to the conventional monolayer cultures and monolayer chondrocyte sheets (Fig. 3B–F).

### Immunohistochemistry

**Figure 4:**
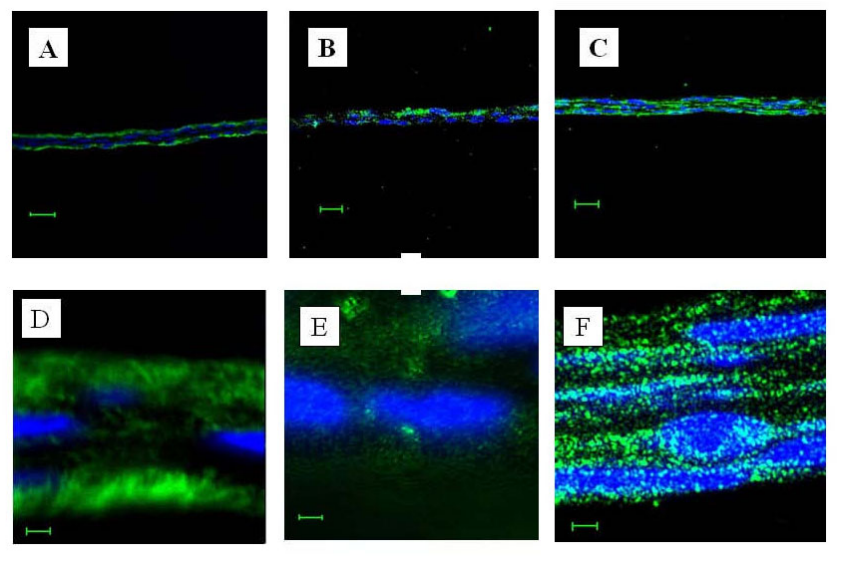
**Immunohistochemical examination: fluorescence microscopy of layered chondrocyte sheets**. Fibronectin (shown in green) was detected in the periphery of the triple-layered chondrocyte sheets (A, D). However, collagen type II (shown in green) was located in the pericellular matrix of the triple-layered chondrocyte sheets (B, E) and integrin α10 (shown in green) was scattered diffusely throughout the triple-layered chondrocyte sheets (C, F). The blue color shows counterstained DNA. DAPI excites at about 360 nm and emits at about 460 nm when bound to DNA, producing a blue fluorescence. A, B, C: Scale bar = 20 μm. D, E, F: Scale bar = 2 μm

**Figure 5:**
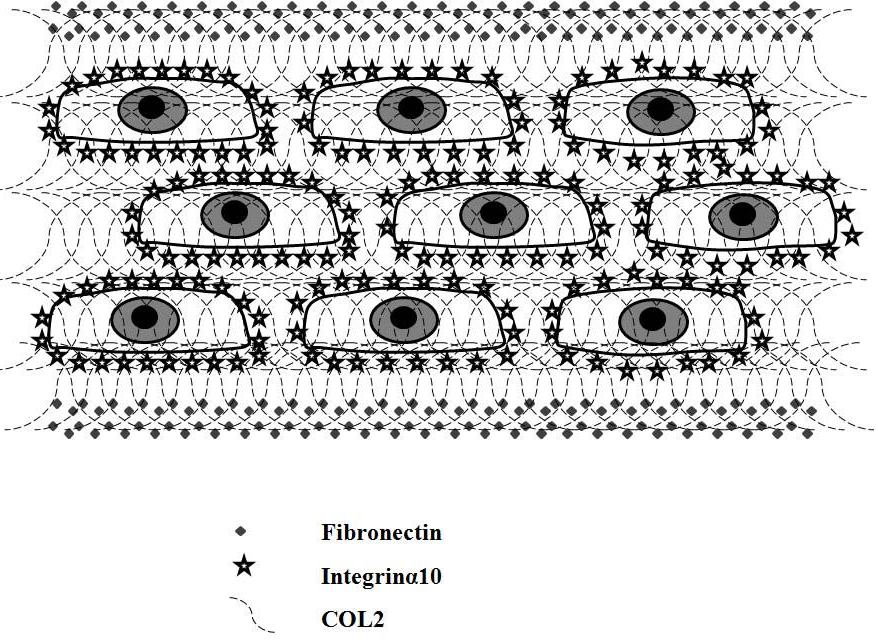
**Schematic illustration of the distribution pattern of extracellular adhesion molecules in triple-layered chondrocyte sheets**. Fibronectin was located peripherally in the triple-layered chondrocyte sheet and integrin α10 was observed close to the chondrocytes, in the pericellular matrix, in the layered construct. However, collagen type II was scattered diffusely throughout the layered cell sheets.

Immunohistochemical examination revealed that fibronectin, integrin α10, and COL2 were present in the triple-layered chondrocyte cell sheet (Fig. 4). Interestingly, fibronectin was located in the periphery of the triple-layered chondrocyte sheets. (Fig. 4A, D) and COL2 was observed in the pericellular matrix of the triple-layered chondrocyte sheets (Fig. 4B, E). However, in contrast to these two proteins, integrin α10 was diffusely distributed throughout the triple-layered chondrocyte sheets (Fig. 4C, F). These different immunohistochemical features of target proteins are illustrated in Fig. 5, where the main results are presented together.

## Discussion

Cell-sheet technology using temperature-responsive culture dishes was first reported by Okano *et al*. in 1993.[8] Since their report was published, this technology has been studied with regard to regenerative medicine for the cornea, heart, pancreas, and liver. [10-13] Nishida *et al*. reported that corneal cell sheets cultured in temperature-responsive culture dishes could strongly adhere to the cornea without scaffolding or suturing.[10] Kushida *et al*. reported that fibronectin expression was preserved on the basal side of cell sheets cultured on temperature-responsive culture dishes.[14] As cell sheets can be harvested with the ECM and adhesion factors, it is simple to layer the cell sheets one on top of another, using the natural adhesiveness of the basal side. Therefore, large layered three-dimensional tissues without a scaffold can be constructed in this repeating fashion. Shimizu et al. reported that the maximum thickness of the fabricated rabbit myocardial cell sheet is three layers in vitro because thicker sheets receive inadequate nutrition. They also demonstrated that repetitive allografts of cell sheets cannot increase the thickness of the fabrication by more than 1 mm in myocardial tissues in vivo. [12]

To fabricate the multilayered sheets, we extended the culture by 1 week, which was effective enough to consolidate the cell sheets into a single three-dimensional structure. However, the cell sheets tended to float in the culture medium because of their shape. Accordingly, it was necessary to devise strategies to apply a physical force to the sheets to enable them to attach gently to each other and the bottom of dish during culture. Using cell-culture inserts of an appropriate height and the weight of the culture-dish cover, we achieved an appropriately narrow space to facilitate strong adhesion between the cell sheets. Although our experimental study of allografts using layered chondrocyte sheets has been proceeding for only 2 months, good adhesion and an inhibitory effect on cartilage degeneration at injured sites has been confirmed (unpublished data).

The SEM examination of the cell sheets indicated that the top and the adhesive basal aspects were completely different in texture. A network of laminated ECM was observed on the top side of the sheets (Fig. 2A). These sheets of ECM structure resemble the lamination of the normal superficial cartilage zone, the "lamina splendens," as initially proposed by MacConaill and later identified by Clark using SEM;[15] however, the scanning electron micrographs show that the sheets do not have a smoother surface than normal articular cartilage.[15] In this study, it was impossible to observe a distinct collagen fibrous structure, which may exist beneath the lamination. According to scanning electron micrographs of ordinary cartilage,[15] in the superficial zone several layers of collagen fibrils exist immediately beneath the *lamina splendens*, forming a mesh of interwoven fibrils that run parallel to the articular surface, and the chondrocytes in this zone appear to be located beneath the layers of collagen fibrils. Meanwhile, on the basal aspect, numerous mound-like elevations were observed in the surface with a texture similar to an aggregation of chondron-like shapes. This smoother surface more resembled normal cartilage surface than did the top side of the cell sheets (Fig. 2B). The flat and smooth surface of the basal aspect implies abundant accumulation of extracellular proteins therein, and is reflected in their characteristic adhesiveness. The flat bottoms of the culture dish and the effects of gravity during the culture period may also fashion this even surface texture.

The concept of the chondron was first introduces by Benninghoff in 1925, with the chondrocyte and its pericellular capsules together representing the chondron, historically considered the primary structural, functional, and metabolic unit of articular and other hyaline cartilages.[16] During recent decades, many researchers have investigated and established the molecular anatomy, functional properties, and metabolic contribution of the chondron in articular cartilage homeostasis and its failure during the initiation and progression of degenerative osteoarthritis. It is interesting that SEM evaluations of the basal aspect of the cell sheets suggest that chondrocytes with ECM, chondrons, were embedded in opposing sides of the sheet surfaces. Although chondrons were only faintly observed because of the thick ECM, it is clear that our sheets contain the basic structural, functional, and metabolic units of articular cartilage and it is expected that they will maintain their function of reduction of friction and the transmission of load. It is thus suggested that, using our technique, these triple-layered chondrocyte sheets have substantially reconstructed the ordinary superficial zone of articular cartilage. To our knowledge, this is the first report of the morphologic evaluation of the bottom aspect of cultivated chondrocytes, which was made possible because the cell sheets were harvested as a single contiguous shape using a noninvasive method without enzyme digestion, thus keeping their original structure.

In this study, the properties of the chondrocyte sheets were investigated, including the expression and localization of SOX9, COL1, 2, 27, integrin α10 and fibronectin. SOX9 has recently been shown to be involved in the control of cell-specific activation of COL2A1 in chondrocytes and to directly regulate the type II collagen gene *in vivo*.[17] Therefore, the high mobility group protein SOX9 is emerging as a key regulator of chondrogenesis. Moreover, Jenkins *et al*. recently reported that the newest cartilage collagen gene, COL27A1, contains two enhancer elements that bind SOX9.[18] Integrin α10 is specifically expressed in chondrocytes. Chondrocytes, depending on the species and tissue origin, express a characteristic set of integrins, including receptors for collagen type II (α1β1, α2β1, and α10β1), fibronectin (α5β1, αvβ3, αvβ5), and laminin (α6β1). Among these receptors, integrin α10β1 is the major integrin mediating chondrocyte-collagen interactions in cartilage.[19,20] Compared with chondrocytes in conventional monolayer culture and single chondrocyte sheets, the significantly higher expression of SOX9 and COL27 mRNA in the layered chondrocyte sheets revealed characteristics more closely resembling normal chondrocyte differentiation, which implies the maintenance of a phenotype. In addition, significantly higher expression of fibronectin and integrin α10 mRNA in the layered chondrocyte sheets also demonstrated the adhesiveness of the cell sheets. Furthermore, on immunohistochemical examination, the expression of fibronectin and integrin α10 in the layered chondrocyte cell sheets verified this adhesiveness and illustrated the specific cartilaginous phenotype of the cell sheets. In both this study and in earlier results using temperature-responsive surfaces[14] it was possible to recover monolayer cell sheets together with deposited fibronectin. Fibronectin matrix adhering to the basal side of cell sheets can function as a glue to attach cell sheets onto other surfaces.[14] In fact, cell sheets recovered from temperature-responsive surfaces easily adhere to other surfaces.[21] Interestingly, as illustrated by the immunohistochemical results in this study (Fig. 5), fibronectin was detected peripherally on both sides of the triple-layered chondrocyte sheets, not only on the basal aspect as described previously[14] but also on the top side, while showing intense adhesiveness on the basal side only. It is possible to hypothesize that the culture period prolonged by 1 week with a cell-culture insert may affect the localization of fibronectin. In this period, the fibronectin could either be moved from its position in the single-layer cell sheets or be newly secreted in areas of contact with the insert. However, the exact mechanism underlying this phenomenon is unclear in our results and further research into this is required.

It is inevitable that for medical applications and when using vital cells such as in ACI, preparation times, including *in vitro *culture periods, will need to be shortened. Overall, it is important to be able to reduce the chondrocyte culture time before cell-sheet harvesting, with reduced time for cell expansion and ECM production, but without changing the extra week of culture to reinforce the cell sheets as this prolonged step is substantially beneficial to this new strategy to cure OA using bioengineered chondrocyte sheets.

In conventional ACI[6] for cartilage regeneration, transplanted periosteal patches, which are used to enclose the implanted chondrocytes, sometimes cause hypertrophy of the regenerated chondral surface. Some improved methods use alternative scaffolds such as collagen membranes, hyaluronan polymers, and atelocollagen gels.[7,22] In comparison to these conventional ACI techniques, the cell-sheet technology has the specific advantage of generating three-dimensional tissues fabricated by autologous cells without using a scaffold while also showing intense adhesiveness on the basal side. The advantages of such cell sheets are that it is easy to culture and expand, and, most importantly, they have good adhesion and barrier function.[9] This means they can protect against intra-articular catabolic factors and prevent proteoglycan escape from the injured site. Moreover, these cell sheets are considered to contain an advantageous supply of growth factors. Furthermore, such cell sheets could be useful as an alternative to the periosteum itself, which is usually used in ACI. Although cell sheets have good adhesive properties compared with other applications using cell-sheet techniques, such as in the cornea and heart, transplanted chondrocyte sheets will be exposed to a harsh environment resulting from weight bearing and friction. Development of new devices to prevent the sheets peeling from the transplanted site is indispensable to future clinical application.

Although focal gene delivery using a cell sheet has not been addressed in this paper, this may also have clinical potential for treatment of cartilage degeneration. The development of a therapeutic apparatus to deliver the cell sheet to the injured site less invasively may therefore be fundamental to expanding its use in the treatment of patients demonstrating the early stages of osteoarthritis.

The results of this study therefore lead to a new strategy for cartilage regeneration using novel bioengineered chondrocyte sheets produced using a cell-sheet technique.

## Conclusion

These experiments demonstrated that triple-layered chondrocyte sheets contain the phenotypic markers COL2, COL27, SOX9, and the adhesion molecules integrin α10 and fibronectin. Cell-sheet technology therefore provides particular advantages for cartilage regeneration, giving three-dimensional tissue constructed without a scaffold and with good adhesiveness to both itself and to an injured cartilage site.

## Methods

### Preparation of human chondrocytes

This study was performed in compliance with the Helsinki Declaration, and was approved by the Institutional Review Board for Clinical Research of Tokai University School of Medicine (ref. 04–056).

Human chondrocytes were obtained from the knee joints of young athletes who underwent anterior cruciate ligament reconstruction at Tokai University Oiso hospital from December 2004 to April 2006. Twenty-nine knees from 29 patients aged 14 to 49 years (21 males and 8 females) were used as the source of these cells. All subjects provided informed consent. The specimens were stored in basal medium (BM) containing Dulbecco's modified Eagle's medium/F12 (DMEM/F12; GIBCO, Invitrogen Corporation, Carlsbad, CA, USA) supplemented with 10% heat-inactivated fetal bovine serum (FBS; GIBCO) and 50 μg/ml ascorbic acid (Wako Pure Chemical Industries, Ltd, Osaka, Japan) and 1% antibiotic-antimycotic mixture (ABAM; 10,000 U/ml penicillin G, 10,000 μg/ml streptomycin sulfate, and 25 μg/ml amphotericin B as Fungizone; GIBCO) until required for the next step. The cartilage samples were cut into small pieces. Thereafter, minced specimens were digested for 1 hr in BM containing 0.4% Pronase E (Kaken Pharmaceutical Inc., Tokyo, Japan), and for a further 4 hrs in BM containing 0.016% Collagenase P (Roche Diagnostics GmbH, Mannheim, Germany). The digested cell suspension was passed through a cell strainer (BD Falcon™; BD Bioscience, Bedford, MA, USA) with a pore size of 100 μm, and the isolated cells rinsed twice with chilled Dulbecco's phosphate-buffered saline (PBS; Dainippon Pharmaceutical Co., Osaka, Japan). The chondrocytes were then seeded into 500 cm^2 ^square dishes (245 × 245 mm; Corning Inc., Corning, NY, USA) at a density of 10,000 cells/cm^2 ^and cultured in BM with 20% FBS (GIBCO) at 37°C in an atmosphere of 5% CO_2 _and 95% air (according to the method of Sato *et al*.).[23]

### Temperature-responsive culture dishes

The specific procedures for the preparation of temperature-responsive culture dishes (provided by CellSeed, Inc) have all been previously described.[24] Briefly, *N*-isopropylacrylamide (IPAAm) monomer solution was spread onto commercial tissue-culture polystyrene dishes. These dishes were then subjected to electron beam irradiation, thus resulting in polymerization and covalent binding of the IPAAm to the dish surface. Poly-IPAAm (PIPAAm)-grafted dishes were rinsed with cold distilled water to remove any ungrafted IPAAm. Finally, the culture dishes were sterilized using ethylene oxide gas.

### Preparation of conventional monolayer cultures of chondrocytes and single-layer chondrocyte sheets

To detach the primary passage cells, chondrocytes were digested using 0.05% trypsin:EDTA (GIBCO), and then counted using a hematocytometer. For the conventional monolayer culture, cartilage cells were seeded into culture dishes (diameter: 35 mm, Iwaki Glass Company, LTD., Tokyo, Japan) at a density of 1,000 cells/cm^2^. To prepare the single-layer chondrocyte sheets, resuspended chondrocytes were seeded at a density of 10,000 cells/cm^2 ^in UpCell culture dishes (diameter: 35 mm, provided by CellSeed, Inc.). The seeded chondrocytes were cultured in BM adjusted to 20% FBS (GIBCO) at 37°C in an atmosphere of 5% CO_2 _and 95% air. At 100% confluence, the cultured cells were harvested and prepared for gene expression analysis.

The samples in conventional culture were harvested with a sterile cell scraper. To release confluent cells as a monolayer chondrocyte sheet from the UpCell temperature-responsive culture dishes, the dishes were removed from the incubator and let stand at about 25°C for 30 min. The culture medium was then removed from the dish, and the cell sheet harvested using polyvinylidene difluoride (PVDF) membrane as a supporting membrane. The lifted chondrocyte sheet edges promptly attached to the overlaid supporting membrane, and the cell sheet and PVDF membrane film were gently detached from the UpCell dish.

### Fabrication of cell sheet into three-layer sheets of chondrocytes

Each cell sheet prepared as above was transferred onto another confluent chondrocyte sheet to fabricate multilayered sheets. Because the multilayered sheets spontaneously floated in culture medium, a 0.4 μm cell culture insert (Falcon, Becton Dickinson, NJ, USA) was placed on top to prevent floating, and then culture of the sheets was continued for 1 week to obtain firm and perfect integration of the cells in the multilayer chondrocyte sheets.

### Scanning electron microscopy evaluation

Triple-layered chondrocyte cell sheets were soaked in 0.1 mol/l phosphate buffer and 2% glutaraldehyde for 2 h. Next, the samples were fixed in 1% osmium solution for 1 h and dehydrated in ascending concentrations of ethanol (50%, 70%, 80%, 90%, 95%, and 100%). The specimens were dried using the critical point drying method, spatter-coated with gold, and affixed to an adhesive interface for observation by SEM (JSM-840; Jeol Ltd., Tokyo, Japan). Both the top and bottom surfaces of the cell sheets were observed.

### RNA isolation and cDNA synthesis

Total RNA extraction was carried out using the RNeasy Mini kit (Qiagen Inc., Valencia, CA) according to the manufacturer's instructions. RNA quality from each sample was determined using the A260/280 absorbance ratio and by electrophoresis on 1.2% agarose formaldehyde gel. Total RNA (1.0–2.0 μg) was reverse transcribed into single stand cDNA using MuLV reverse transcriptase (Applied Biosystems, Foster City, CA, USA). The reverse-transcription reaction was performed in a thermocycler at 42°C for 60 min and then at 95°C for 5 min.

### Primer design and real-time PCR

All oligonucleotide primer sets were designed based upon published mRNA sequences. The expected amplicon lengths ranged from 70 to 200 bp. The oligonucleotide primers used in this study are listed in Table 1. The real-time PCR was performed in a SmartCycler™ (Cepheid, Sunnyvale, CA, USA) using SYBR Green PCR Master Mix (Applied Biosystems). From 2 to 2.5 μl of cDNA template was used for real-time PCR in a final volume of 25 μl. cDNA was amplified according to the following condition: 95°C for 15 s and 60°C for 60 s from 35 to 45 amplification cycles. Fluorescence changes were monitored with SYBR Green after every cycle. A melting curve analysis was performed (a 0.5°C/s increase from 55 to 95°C with continuous fluorescence readings) at the end of cycles to ensure that single PCR products were obtained. The amplicon size and reaction specificity were confirmed by 2.5% agarose gel electrophoresis. All reactions were repeated in six separate PCR runs using RNA isolated from four sets of human samples. The results were evaluated using the SmartCycler™ software program. Glyceraldehyde-3-phosphate dehydrogenase (GAPDH) primers were used to normalize samples. To monitor crossover contaminations of PCR, RNase-free water (Qiagen Inc) was included in the RNA extraction and was used as a negative control. To ensure the quality of the data, a negative control was always included in each run.

**Table 1:** List of primers used in the real-time PCR.

Primer ID	Accesion No.	Sequence	Expect size(bp)
Collagen Type 1-F	NM_000088	AAG GGT GAG ACA GGC GAA CAA	170
Collagen Type 1-R		TTG CCA GGA GAA CCA GCA AGA	

Collagen Type II-F	NM_033150	GGA CTT TTC TTC CCT CTC T	113
Collagen Type II-R		GAC CCG AAG GGT CTT ACA GGA	

SOX9-F	NM_000346	AAC GCC GAG CTC AGC AAG A	138
SOX9-R		CCG CGG CTG GTA CTT GTA ATC	

Collagen27α1-F	NM_032888	GGG CCT TAT GGA AAT CCA GGT C	176
Collagen27α1-R		GGT CCA GGA TAG CCC TTG TGT C	

Integrinα10-F	NM_003637	CTG GGA TAT GTG CCC GTG TG	112
Integrinα10-R		TTG GAG CCA TCC AAG ACA ATG A	

Fibronectin1-F	NM_001030524	GCA CAG GGG AAG AAA AGG AG	189
Fibronectin1-R		TTG AGT GGA TGG GAG GAG AG	

### Immunohistochemical staining

Frozen sections (30 × 24 × 5 mm) of triple-layered cell sheets were prepared using OCT compound (Sakura Fine Technical Co., Tokyo, Japan). The sections were then washed in PBS, and were reacted at room temperature for 60 min with three monoclonal antibodies: an anti-fibronectin mouse monoclonal antibody (clone FBN11, diluted 1:500, #MS-1351-P0; Thermo SCIENTIFIC, Lab Vision Co., CA, USA), an anti-human CD11c (integrin α10) mouse monoclonal antibody (clone BU15, diluted 1:200, #SM1834PS; Acris Antibodies GmbH, Herford, Germany), and an anti-human collagen type 2 mouse monoclonal antibody (clone α-4C11, diluted to 5 μg/ml, #F-57; Daiichi Fine Chemical Co., Toyama, Japan). The sections were washed in PBS, and reacted with polyclonal rabbit anti-mouse immunoglobulin/FITC, diluted 1:100 (#F0261; DAKOcytomation, Glostrop, Denmark) as a fluorescent secondary antibody. Mounting with a water-soluble mounting medium (VECTASHIELD^® ^Mounting Medium with DAPI, Vector Laboratories, Inc., Burlingame, CA, USA) was performed to counterstain DNA after washing sections in purified water.

### Statistical analysis

The real-time PCR results are expressed as the mean ± standard error of the mean from six determinations and representative results are shown. The statistical software program SPSS (Version 17.0, SPSS, Chicago, IL, USA) was used to perform standard analysis of variance and the Scheffe's post hoc test. Table 2 lists the p-values shown in Figure 3.

**Table 2:** Results of the post hoc test (Scheffe's method).

Dependent variable	(I) V1	(J) V1	Difference of averages (I-J)	SEM	P-value	95% CI		Figure 3
						Lower limit	Upper limit	
COL1	Mono	Cell Sheet	.05250	.05374	.635	-.1043	.2093	
		
		Layered	.39500*	.05374	.000	.2382	.5518	*1
	
	Cell Sheet	Mono	-.05250	.05374	.635	-.2093	.1043	
		
		Layered	.34250*	.05374	.000	.1857	.4993	*2
	
	Layered	Mono	-.39500*	.05374	.000	-.5518	-.2382	*1
	
		Cell Sheet	-.34250*	.05374	.000	-.4993	-.1857	*2

COL2	Mono	Cell Sheet	-.12750	.05183	.099	-.2787	.0237	
		
		Layered	-.52500*	.05183	.000	-.6762	-.3738	*3
	
	Cell Sheet	Mono	.12750	.05183	.099	-.0237	.2787	
		
		Layered	-.39750*	.05183	.000	-.5487	-.2463	*4
	
	Layered	Mono	.52500*	.05183	.000	.3738	.6762	*3
		
		Cell Sheet	.39750*	.05183	.000	.2463	.5487	*4

SOX9	Mono	Cell Sheet	.04500	.08833	.880	-.2127	.3027	
		
		Layered	-.68500*	.08833	.000	-.9427	-.4273	*5
	
	Cell Sheet	Mono	-.04500	.08833	.880	-.3027	.2127	
		
		Layered	-.73000*	.08833	.000	-.9877	-.4723	*6
	
	Layered	Mono	.68500*	.08833	.000	.4273	.9427	*5
		
		Cell Sheet	.73000*	.08833	.000	.4723	.9877	*6

COL27	Mono	Cell Sheet	-.13250	.17224	.751	-.6350	.3700	
		
		Layered	-.43750	.17224	.088	-.9400	.0650	*7
	
	Cell Sheet	Mono	.13250	.17224	.751	-.3700	.6350	
		
		Layered	-.30500	.17224	.260	-.8075	.1975	
	
	Layered	Mono	.43750	.17224	.088	-.0650	.9400	*7
		
		Cell Sheet	.30500	.17224	.260	-.1975	.8075	

Integrin a10	Mono	Cell Sheet	.05500	.10949	.883	-.2645	.3745	
		
		Layered	-.36500*	.10949	.027	-.6845	-.0455	*8
	
	Cell Sheet	Mono	-.05500	.10949	.883	-.3745	.2645	
		
		Layered	-.42000*	.10949	.013	-.7395	-.1005	*9
	
	Layered	Mono	.36500*	.10949	.027	.0455	.6845	*8
		
		Cell Sheet	.42000*	.10949	.013	.1005	.7395	*9

Fibronectin	Mono	Cell Sheet	-.71500*	.16992	.007	-1.2108	-.2192	*10
		
		Layered	-.93000*	.16992	.001	-1.4258	-.4342	*11
	
	Cell Sheet	Mono	.71500*	.16992	.007	.2192	1.2108	*10
		
		Layered	-.21500	.16992	.479	-.7108	.2808	
	
	Layered	Mono	.93000*	.16992	.001	.4342	1.4258	*11
		
		Cell Sheet	.21500	.16992	.479	-.2808	.7108	

## Authors' contributions

GM, MS, NK, MK, and TK performed the research. JIL, MI, HS, and JM analyzed the data. HS provided the temperature-responsive culture dishes for these experiments. NO took charge of the statistical analyses. GM, JIL, MS, and JM wrote the manuscript. All authors have read and approved the final manuscript.
